# Exploring the role of ICT in pharmaceutical supply chain practices and operational performance in Ethiopia: a structural equation modeling approach

**DOI:** 10.1186/s12913-023-09627-w

**Published:** 2023-06-14

**Authors:** Rabira Hailu, Tafesse Gizaw, Nimona Berhanu, Tidenek Mulugeta, Bekele Boche, Tadesse Gudeta

**Affiliations:** 1 Guder Primary Hospital, West Shoa, Oromia, Ethiopia; 2 Last Mile Project, Cordaid Ethiopia, Jimma, Ethiopia; 3 Department of social and administrative pharmacy, Institute of Health, Jimma University, Jimma, Ethiopia

**Keywords:** Supply chain practice, Operational performance, ICT, Structural equation modeling, Ethiopian pharmaceutical supply agency

## Abstract

**Background:**

A well-coordinated supply chain ensures the sustainable availability of life-saving medicines that improve public health outcomes. Information Communication Technology (ICT) is one of the strategies for optimizing supply chain coordination. However, there is a paucity of data on how it affects supply chain practice and performance at the Ethiopian Pharmaceutical Supply Agency (EPSA).

**Objective:**

This study aimed to explore the relationships between information and communication technology, supply chain practice, and pharmaceutical supply chain operational performance using a structural equation modeling approach.

**Methods:**

We conducted an analytical cross-sectional study between April and June 2021. Three hundred twenty EPSA employees participated in the survey. We used a pretested, self-administered five-point Likert scale questionnaire to collect the intended data. A structural equation modeling confirmed the relationship between the constructs (information communication technology, supply chain practices, and performance). Thus, the measurement models were first validated using exploratory and confirmatory factor analysis in SPSS/AMOS software. A p-value of less than 5% indicated statistical significance.

**Results:**

Of the 320 questionnaires distributed, 300 participants (202 males and 98 females) duly responded. In this survey, supply chain practices (mainly customer relationship management and information sharing) and ICT had significant positive direct effects on operational performance with standardized regression weights (β) of 0.65 (p < .001) and 0.29 (p < .001), respectively. On the other hand, 73% of the variations in operational performance were explained by ICT and supply chain practices, wherein ICT played moderate mediation effects between supply chain practice and performance (VAF = 0.24, p < .001). Despite the significant positive influence of ICT, the agency still faced data visibility problems with customers and other supply chain partners.

**Conclusion:**

The findings revealed that supply chain practices and ICT implementation impacted the agency’s supply chain performance positively and significantly. The ICT implementation practice in the agency posited a significant positive partial mediating role between supply chain practice and operational performance. Thus, if the agency focuses on the automation and integration of customer relationship management and the practice of information exchange, the essential supply chain practices, it can further improve operational performance.

**Supplementary Information:**

The online version contains supplementary material available at 10.1186/s12913-023-09627-w.

## Introduction

Supply Chain Management (SCM) is a multi-functional activity encompassing operations ranging from demand creation to order fulfillment. Its goal is to ensure the seamless flow of goods, data, and finance between channel partners, resulting in increased productivity and efficiency while lowering associated costs [1]. It has applications in various areas. In the healthcare sector, Pharmaceutical Supply Chain Management (PSCM) refers to the stakeholders, systems, and processes required for the pharmaceutical flow from manufacturers to the patient’s bedside or use. Its ultimate purpose is to guarantee that the appropriate goods are available to support patient care in the appropriate quantities and locations [2].

A robust PSCM system ensures a sustainable availability of life-saving medicines and other health technologies to successfully achieve global goals of halting the HIV/AIDS epidemic, eradicating tuberculosis and malaria, preventing maternal and child mortality, and improving overall public health outcomes [3]. People seeking treatment rely on an uninterrupted supply of essential medicines to maintain or restore body functions. Thus, a well-coordinated Pharmaceutical Supply Chain Management (PSCM) has an enormous impact on a company’s operational performance [4].

In PSCM, operational performance refers to the extended supply chain activities leveraged to achieve end-customer expectations such as product availability, optimal cost, service quality, delivery time and reliability, and flexibility in providing services [5, 6].

In daily operations, many organizations strive to enhance their supply chain performance through optimized supply chain practices like technological improvements [7, 8]. It can be accomplished by integrating information and communication technology (ICT) into fundamental supply chain activities [9, 10]. ICT in SCM is crucial in refining the flow of supply chain decisions to achieve organizational competitiveness, enhance service level, improve inventory visibility, fasten transaction execution, and facilitate collaboration and coordination among supply chain partners. Decision-makers may plan, manage, and adapt operations to assemble in procurement, inventory, or manufacturing using real-time information about the various phases of the supply chain network. Indeed, a supply chain can only function if it is properly integrated and coordinated through the adoption of ICT tools or systems [11]. For example, due to COVID-19, substantial industrial sectors have been affected globally by curfew labor shortages and were struggling to stay productive by adopting automated systems and the Internet of Things (IoT) technologies that improved connectivity in supply chains [12]. Similarly, reports from Kenya and Uganda revealed the remarkable role of digital technology in the pharmaceutical industry in streamlining pharmaceutical SCM processes through improving procurement systems, ordering, information tracking, and communication and coordination practices [13].

In Ethiopia, the Pharmaceutical Supply Agency (EPSA) is the only public agency mandated to ensure a sustainable supply of medicines and serves as the source of pharmaceuticals throughout the country. Currently, the EPSA performs pharmaceutical quantification, procurement, warehousing, and distribution activities to supply public health facilities [14, 15]. It has heavily invested in information communication technology (ICT) in recent years as part of its efforts to improve supply chain management; for example, the implementation of an Integrated Health Commodity Management Information System (HCMIS), which uses a central database to manage pharmaceutical stock levels in multiple warehouses across the country. It also uses an electronic Logistics Management Information System (e-LMIS) to track drug orders and deliveries among stakeholders in the medical value chain. In addition, the agency completed the preliminary preparation for the Enterprise Resource Planning (ERP) system. With these technologies and other value-added processes, it aspires to fulfill its mission of ensuring a sustainable supply of quality-assured medicines to the public [16].

Despite EPSA’s remarkable work, internet access and equipment, shortage of skilled workers, and power outages continue challenging the upstream and downstream supply chain, leading to poor data quality and information sharing (weak coordination), wastage, or shortages of medicines [17–21]. Evidence from a systematic review between 2003 and 2019 shows that the average availability of essential medicines in Ethiopia is only 75% [22]. On the other hand, EPSA’s total pharmaceutical expenditure grows continuously. During the same time frame, the purchase value of the Revolving Drug Fund and program commodities increased by about 35%, from USD 293,120,000 in 2016 to USD 452,760,000 in 2019 [14].

Although few studies are available on supply chain practices [23–25] and performance [26–30], there is little information on the role of digital technology in the healthcare supply chain in Ethiopia. The few available studies, e.g., [17, 31], are descriptive and measure outcomes of practices. They do not show the extent and direction of the impact of ICT on supply chain practices, and it is uncertain whether the performance is due to ICT or other initiatives because the health supply chain system is undergoing transformations. Thus, it is crucial to model and investigate the relationships between the three critical variables, i.e., information and communication technology, supply chain practices, and operational performance, using advanced statistical modeling, and distinguish priority areas for efficient allocation of resources. The present study applied structural equation modeling to quantitatively determine the interrelationships between the predictors (supply chain practices), mediator (ICT), and outcome (operational performance) variables. Thus, it provides insights into practical approaches that developing countries with similar resources and logistical hassles can undertake to improve their pharmaceutical management capability while increasing productivity rates at various stages.

## Methods

### Study settings and period

We conducted the study at the EPSA head office and selected branches between April and June 2021. The EPSA head office in Addis Ababa [Ethiopia’s capital] coordinates the activities of 19 EPSA branches. The head office procures, stores, and distributes healthcare products to the branches. The branches are in the charge of distributing and quantifying healthcare products to over 4,000 health facilities. The agency had an adequate number and mix of professionals, including medical doctors, pharmacists, druggists, laboratory professionals, biomedical engineers, accountants, ICT officers, and others with different experiences. As a sole public supplier, EPSA implemented several initiatives, and it has a plan to excel in its supply chain services with a vision of “the most responsive and efficient pharmaceuticals supply chain organization in Africa by 2030” through enhancing customer service, operations, financial sustainability, leadership, governance, and human resource management [14]. Thus, studies, including the present one, are valuable inputs for realizing its vision.

**Study design**: The study was a quantitative survey based on an analytical cross-sectional design to determine the relationships between information and communication technology, supply chain practices, and operational performance using Structural Equation Modeling (SEM). SEM consists of two sub-models: measurement and structural. The measurement model quantifies the latent (unobserved variables) while the structural model tests hypotheses based on path analysis [32].

### Population and sampling procedures

The EPSA head office, all branches, and their personnel contained the source population. The EPSA head office, the chosen branches, and employees whose jobs were directly connected to PSCM in the agency comprised the study population. We sampled three branches based on the logistics indicators assessment tool’s recommendation of taking at least 15% of total facilities [33]. We chose the EPSA head office on purpose since it is at the epicenter of the pharmaceutical supply chain, both upstream (manufacturers/suppliers) and downstream (EPSA branches). Adama and Addis Ababa (No. 1) were chosen randomly. We then considered volunteer professionals (directors and supply chain officers of the agency) who work directly with supply chain processes or systems. On the other hand, employees who did not want to participate and were absent during data collection were excluded. Finally, we distributed the questionnaires to 320 employees, i.e., 172 from EPSA headquarters, 86 from Adama, and 62 from Addis Ababa (No. 1) branches.

### Data collection procedures

We used a self-administered structured questionnaire to collect the required data. The questionnaire had four parts. Part I assessed the socio-demographic characteristics of respondents. Part II contained items on supply chain performance using five sub-constructs. Part III had four sub-constructs to measure PSCM practices. Part IV consisted of questions about the ICT implementation practice of the agency. Parts II-IV used agreement-type questions on a five-point Likert scale (1 to ‘strongly disagree’ to 5 to ‘strongly agree’). We developed 42 measuring items (observed variables) from the previous literature. They were then split into eleven categories based on the latent variables they intended to measure. The latent variables were further organized to evaluate the predictor, mediating, and dependent variables. Accordingly, supply chain quality, cost, responsiveness, operational flexibility, and customer satisfaction were sorted to assess the dependent variable, supply chain performance. To measure the independent variable (supply chain practices), we used sub-constructs such as strategic suppliers’ partnerships, customer relationship management, the status of information sharing, warehouse and inventory management, and strategic outsourcing. The two constructs, the level of ICT usage and staff ICT skill enhancement, are used to determine the mediating variable (ICT implementation practice). We recruited four data collectors with a pharmacy background and relevant work experience to gather data from the agency’s officers, directors, and team coordinators.

### Data quality assurance

The questionnaires were developed through an extensive review of previous literature and evaluated by experts to ensure content and face validity. We conducted a pretest at the EPSA Jimma to assess the tool’s applicability and understandability. We performed a reliability test and modified the items for Cronbach’s alpha threshold of less than 0.7 [34]. As a result, 11 items were found to be non-reliable and thus removed, leaving 31 with alpha values ranging from 0.703 to 0.894 for the actual data collection (Additional file 1). The data collectors had a pharmacy background with service years of at least a year in logistics services at health facilities or suppliers. We trained them for one day in the overall data collection process and the purpose of the study.

### Data processing, statistical analysis, and presentation

We used EpiData software (version 3.0) to clean and manage data and analyzed it using statistical software (SPSS and AMOS version 23). To ensure the clarity and simplicity of the structure, the fifteen items used to construct the operational performance were averaged into five components (cross-ponding latent variables), reducing the number of total items from 31 to 21. We summarized the data using both descriptive and inferential statistics. Socio-demographic variables were analyzed using frequency and percentage. We used structural equation modeling (SEM) to test the relationship between the variables. Before running the statistical analysis, we tested the common underlying assumptions, such as normality, linearity, multicollinearity, and homoscedasticity. The data normality was checked by looking at skewness and kurtosis values after filtering out significant multivariate outliers (p < .001) with a Mahalanobis distance. The cut points for absolute Z skewness must be greater than two, and the kurtosis value must be greater than seven [35, 36]. Thus, skewness and kurtosis values are within the normal range and did not violate the normality assumption.

We inspected the scatter plot of residuals to examine the linearity between variables. The graph featured a diagonal line with a positive slope, indicating a positive linear relationship between the dependent and independent variables [37, 38]. To test for multicollinearity among predictor variables, tolerance and VIF (Variance Inflation Factor) statistics with cut-off points of > 0.2 and < 3, respectively, were used [38]. The findings confirmed that there were no multicollinearity issues between the variables. To depict homoscedasticity, we used a standardized residual plot versus projected values in scatter plots to show an even distribution of error terms across all variables. In the study, each predictor variable had nearly the same distribution of standardized error from the predicted variable, implying that the homoscedasticity condition was not violated.

Correlation analysis determines the magnitude and direction of the linear relationships between variables. It also identifies variables with significant relationships and insignificant variables to exclude from regression analysis [39]. As a result, we performed Pearson’s product-moment correlation coefficient (r) between the variables.

After the assumption tests, we run Exploratory Factor Analysis (EFA) to extract variables with eigenvalues greater than one and factor coefficients > 0.4 based on principal component analysis [40]. The suitability of data to proceed with EFA was checked by examining the correlation matrix and KMO (Kaiser-Meyer-Olkin), the measure of sampling adequacy. An orthogonal varimax rotation with Direct Oblimin was used to ascertain whether the correlation between any two factors surpassed the 0.32 cut-off point (i.e., more than 10% overlap in variance between variables). We performed Confirmatory Factor Analysis (CFA) to ensure that the data fit the model after removing items that loaded on two components and those with low factor loadings (< 0.4) [41]. We validated the measurement model by assessing the reliability and validity tests before evaluating the structural relationships among latent variables [32]. The reliability test indicates the internal consistency of measured variables using Cronbach’s α statistics and composite reliability. Composite reliability assesses the extent to which the indicator variables converge and share a proportion of the variance. It provides a less biased assessment of reliability than Cronbach’s alpha. The acceptable range of values should be greater than 0.70 [42, 43].

Validity reveals how well a measure reflects its unobservable factors. Thus, testing the relationships between measured items and constructs and the relationships between constructs is a way to confirm validity [43]. In this study, we assessed the convergent and discriminant validity using confirmatory factor analysis in AMOS software. Convergent validity examines how well a measure correlates with other measures of the same construct or with other latent variables. It quantifies the degree to which measurement items converge on a theoretical notion. Convergent validity is usually evaluated by strong bivariate correlations, significant factor loadings, and average variance extracted (AVE). The value of AVE—the sum of squared standardized factor loadings divided by the number of items—should be greater than or equal to 0.5 to support convergent validity [44].

Discriminant validity indicates that one construct is unique from another and has a high variance with its items as opposed to items from other constructs. The square root of the AVE must be greater than the correlation among latent variables to prove to construct distinctness. Constructs with correlations less than 0.70 also indicate construct distinctness [44]. The maximum likelihood estimation technique in AMOS software was used to validate the goodness of data to the model. We used confirmatory factor analysis to validate the measurement model by utilizing multiple fit indices. The most commonly used model fit indices and their conventional cut-off values are > 0.90 (GFI, CFI, AGFI, NFI, TLI, and RFI), RMSEA < 0.50, and CMIN/df < 5.0 or p-value > 0.05 [45, 46]. Following the validation of the measurement model, we conducted a structural path analysis to examine the strength of relationships between constructs. We tested the hypotheses using standardized path coefficients (β) and p-values.

We performed a mediation analysis (ICT implementation practice in the relationship between PSCP and operational performance). The mediation effect size was determined using the web-based Sobel test. In this test, Variation Accounted For (VAF) determines how much of the direct path is absorbed. It is the ratio of indirect effects to total effects. The rule of thumb is that if VAF is less than 0.2, it implies no mediation; VAF between 0.20 and 0.80 signifies partial mediation; and VAF greater than 0.80 means complete mediation [44, 47]. We presented the findings in tables, charts, and structural equation models.

## Results

### Socio-demographic characteristics

Of the 320 distributed questionnaires, 304 respondents completed and returned them, with a response rate of 95%. However, four cases were aberrant and excluded, leaving 300 for final analysis. Male respondents constituted the most, representing 202 (67.33%). The majority, 91 (30.33%), were aged between 36 and 45. Two hundred seventeen (72.34%) had a bachelor’s degree, and 163 (54.33%) had 6 to 10 years of work experience. One hundred sixteen (38.67%) worked under the warehouse and inventory management unit (Table 1).

**Table 1:** Socio-demographic characteristics of the respondents (n = 300)

Variables	Items	Frequency (%)
Gender	Male	202 (67.40)
	Female	98 (32.60)
Age (years)	< 25	5 (1.67)
	26–35	128 (42.67)
	36–45	141 (47)
	> 46	26 (8.66)
Level of education	Diploma	34 (11.33)
	BSC/BA degree	217(72.34)
	MSc/MA	49 (16.33)
Work experience in the agency (years)	1–5	98 (32.67)
	6–10	163 (54.33)
	> 10	39 (13.00)
The department currently working in	WIM	116 (38.67)
	Distribution and fleet management	60 (20.00)
	Quantification, forecasting, and market shaping	72 (24.00)
	Tender management	13 (4.33)
	Contract management and procurement team	30 (10.00)
	Others*	9 (3.00)

Notes: *project coordinator, branch managers, deputy branch managers, organizational change management team, and planning, monitoring, and evaluation team

## Correlation analysis

Table 2 indicates that all constructs significantly correlated with supply chain operational performance, with a minimum correlation coefficient (r) of 0.374. The results also revealed a significant moderate correlation (r = .333 to 566) between predictor variables, except ICT and WIM, which had a very weak correlation (r = .240).

**Table 2:** The bivariate correlation coefficient between the study variables

Factors	CRM	IQSP	WIM	ICT
CRM	1			
IQSP	0.505^**^	1		
WIM	0.374^**^	0.471^**^	1	
ICT	0.566^**^	0.333^**^	0.240^*^	1
Operational performance	0.637^**^	0.580^**^	0.374^**^	0.696^**^

Notes: CRM = customer relationship management, IQSP = status of information sharing, WIM = warehouse and inventory management, **p-value < 0.001, *p-value < 0.05

### Exploratory factor analysis

In the EFA, we excluded five items (four with loadings less than 0.4 and one with a significant cross-loading) from subsequent analysis. Indeed, five factors with eigenvalues greater than one were extracted. The factor loading for the remaining 16 items ranged from 0.608 to 0.851, which is more than the suggested threshold of 0.50. The five factors explained 67.33% of the variation in the dataset. The KMO (Kaiser-Meyer-Olkin) value of 0.843 and Bartlett’s Test of Sphericity, p < .001, indicated acceptable sampling size and the presence of significant (different from zero) correlations between the variables (Table 3 and Additional file 2).

**Table 3:** Exploratory factor analysis of the study variables

Study variables	Number of items	Factor loadings	Eigen values	% CVE*
ICT implementation	4	[0.651–0.851]	5.292	15.998
Operational performance	4	[0.608–0.730]	2.012	29.823
Status of information sharing	3	[0.741–0.829]	1.335	43.062
Warehouse & inventory mgt practice	3	[0.792-0.800]	1.114	56.020
Customer relationship management	2	[0.793–0.803]	1.020	67.333

Kaiser-Meyer-Olkin (KMO) Measure of Sampling Adequacy = 0.843

Bartlett’s Test of Sphericity (app. Chi-square = 1761.713, *df* = 120, and sig. = 0.000)

Extraction Method: Principal Component Analysis. Rotation Method: Varimax with Kaiser Normalization

Notes: *cumulative variance explained

### Confirmatory factor analysis

The composite reliability of all factors was higher than 0.70, indicating internal consistency. Except for operational performance, the remaining four constructs had an AVE value greater than 0.5. The loading values for each measuring item were more than 0.5, and the construct correlations were statistically significant (p < .001). Thus, though one construct had an AVE value of less than 0.5, the models demonstrated convergent validity. Aside from the correlation between operational performance and ICT (r = .696), no other pair of correlations exceeded the square root of the AVEs, demonstrating discriminant validity (Table 4).

**Table 4:** The measurement models’ reliability and validity with their factors

Factors	OP	ICT	WIM	CRM	IQSP	α -value	CR	AVE
OP	**0.650**					0.708	0.701	0.423
ICT	0.696٭٭	**0.733**				0.818	0.787	0.538
WIM	0.374٭٭	0.240٭٭	**0.723**			0.760	0.764	0.523
CRM	0.637٭٭	0.566٭٭	0.374٭٭	**0.824**		0.808	0.808	0.678
IQSP	0.580٭٭	0.333٭٭	0.471٭٭	0.505٭٭	**0.764**	0.799	0.807	0.584

Note: **p-value < 0.01, diagonal bold numbers represent the square root of AVE (average variance extracted) for each factor, α = Cronbach alpha, CR = Composite reliability

**Fig. 1:**
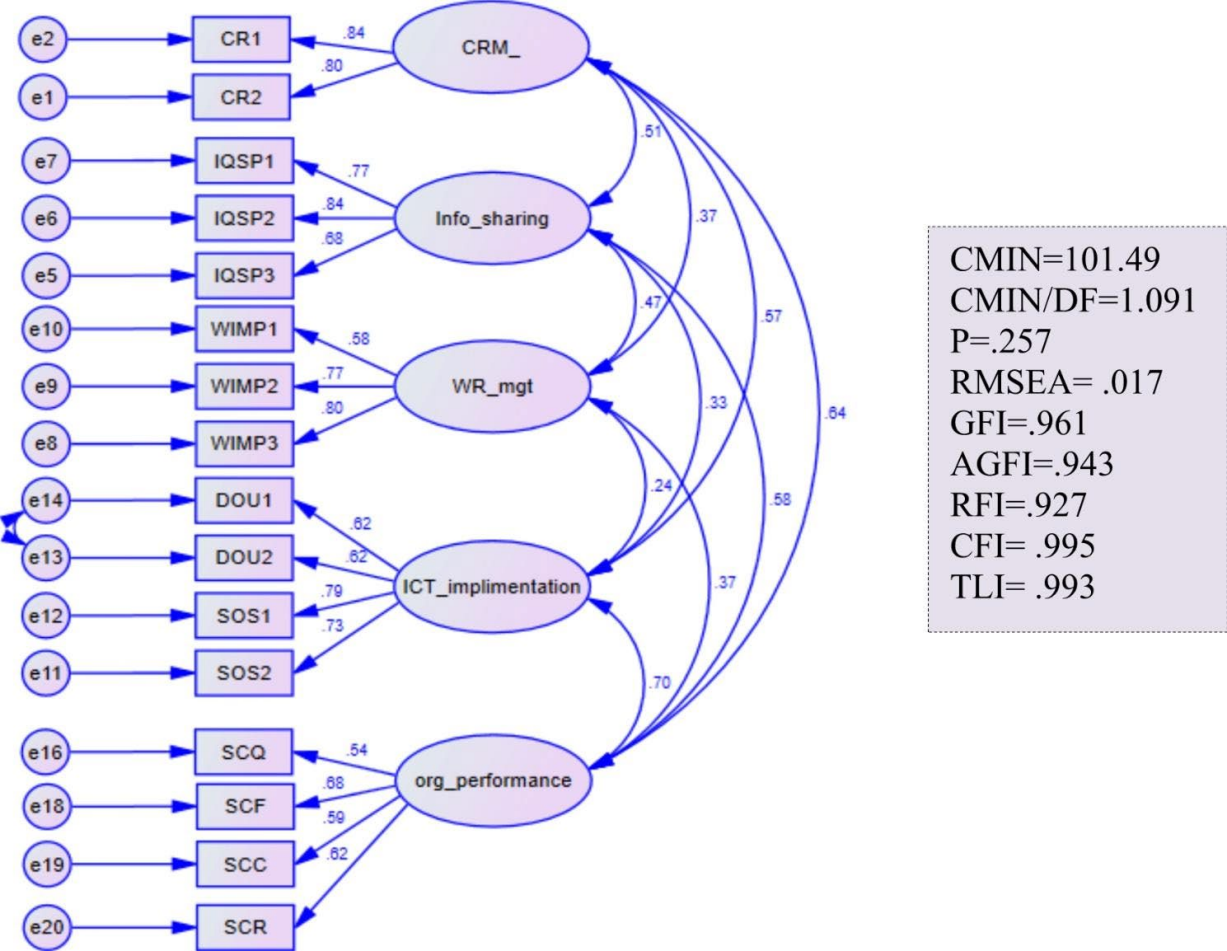
Confirmatory factor analysis of the study variables

As shown in Fig. 1, the observed data and measurement models were in perfect agreement with all goodness of fit indices in the normal range (χ2/df = 1.091, p = .257, RMSEA = 0.017, GFI = 0.961, AGFI = 0.943, CFI = 0.995, RFI = 0.927, and TLI = 0.993). The standardized regression weights between each element and the corresponding latent variable were positive and significant at p < .001. As a result, the proposed measurement models had construct validity.

### Structural path analysis and hypotheses

**Fig. 2:**
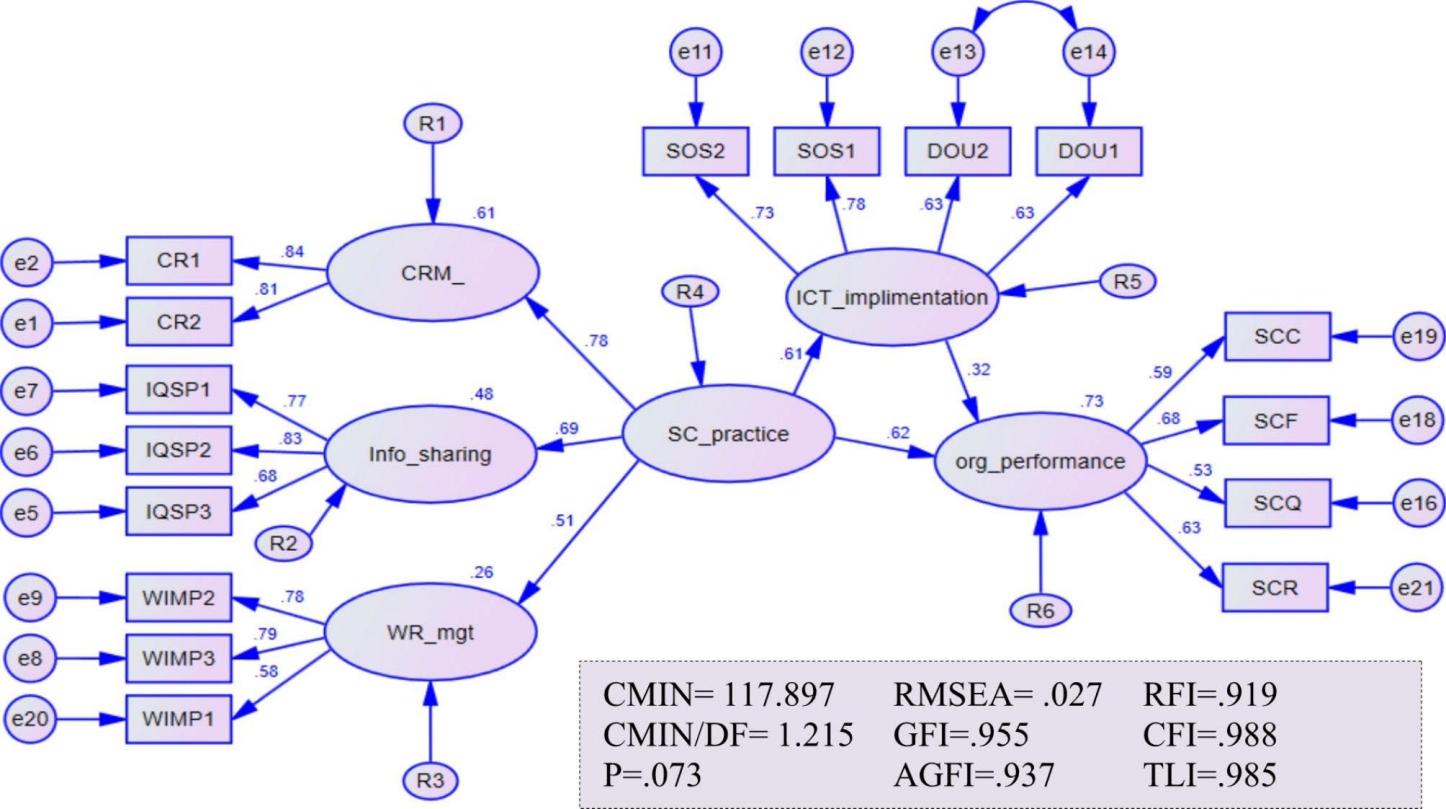
Hypothesized structural path analysis

We constructed the structural model (path analysis) based on modified measurement models. The model fit indices (RMSEA = 0.027, GFI = 0.955, AGFI = 0.937, RFI = 0.919, CFI = 0.988, TLI = 0.98, and CMIN/DF = 1.215) and significant standardized regression weights at p < .001 indicate that the proposed model completely matches the sample data (Fig. 2; Table 5). As a result, the data underpin the structural model and the underlying notion. With R^2^ = 0.73, pharmaceutical supply chain practices (PSCP) and the degree of ICT implementation explained 73% variances in operational performance.

### The hypotheses test results

*H*_*1*_: *Supply chain practices, including warehouse & inventory management practice (WIM), the level of information sharing (IQSP), and customer relationship management (CRM), had a positive relationship with the operational performance of the agency.*

A significant regression coefficient (unstandardized score = 0.52, t-value = 5.04, p < .001) indicates that pharmaceutical supply chain practices (PSCP) had a significant direct positive effect on operational performance. In other words, an additional improvement of the PSCP by one unit, while other factors remain constant, increases operational performance by 0.52 units (Table 5). Based on Fig. 2, all PSCP constructs had standardized regression weights greater than 0.371, indicating strong effect sizes between each construct and PSCP. In contrast, customer relationship management had the highest predictive power, with a standardized estimate (β) of 0.78 at p < .001. Improving CRM by a unit, for example, by measuring customer satisfaction (CR1) and incorporating the results into future business planning (CR2), improves supply chain practices by 0.78.

*H*_*2*_: *Implementation of information communication technology (operationalized with skills of the staff/end-users and degree of ICT usage by end users) has a positive relationship with the organizational performance of the agency.*

From Table 5, the ICT implementation affects the operational performance positively and significantly (B = 0.22, t-value = 3.26), supporting the proposed hypothesis (H2). As shown in Fig. 2, staff development (SOS1), the presence of ICT experts (SOS2), the use of high storage capacity databases (DOU1), and the existence of user-friendly ICT tools (DOU2) were items retained after extensive quality verification and explained the ICT implementation positively and significantly with standardized regression estimates (β) of 0.78, 0.73, 0.63 and 0.63, respectively at p < .001. Of the above dimensions, staff development and the presence of ICT experts highly predicted the agency’s ICT implementation practice. At least one unit increase in trained staff and ICT experts improves the implementation status by at least 0.70.

*H*_*3*_: *Information and communication technology implementation practice has a positive mediation effect on the relationship between supply chain practice and the operational performance of the agency*.

The unstandardized estimate of the direct path from PSCP to operational performance was statistically significant (B1 = 0.52, t-value = 5.04, p < .001). The product of the regression weights from PSCP to ICT and from ICT to operational performance resulted in an indirect effect size of 0.1672, and the path is significant (p < .001). The sum of the direct (B1) and indirect (B2) effects yields the total effect (B = 0.6872). The VAF (the ratio of indirect to total effect) approximated 0.24, which is statistically significant (p < .001). Based on the VAF value of 0.240, ICT implementation practice in the agency posited a partially mediating role in the relationships between PSCP and operational performance, and the hypothesis (H3) was accepted.

**Table 5:** Unstandardized regression estimates of the study variables (PSCP, ICT, & operational performance)

Path	Estimates	S.E.	t-value.	P-value
ICT implementation<--- SC practice	.76	.114	6.70	***
Operational performance<---ICT implementation	.22	.067	3.26	***
Operational performance <--- SC practice	.52	.103	5.04	***
CRM practice<--- SC practice	1.00	Reference (constrained path)		
Status of information sharing <--- SC practice	.82	.116	7.08	***
WIM practice<--- SC practice	.53	.093	5.66	***
DOU1<--- ICT implementation	.76	.081	9.34	***
DOU2<--- ICT implementation	.84	.088	9.55	***
SOS1<--- ICT implementation	1.00	Reference (constrained path)		
SOS2<--- ICT implementation	0.93	0.085	10.97	***
SC flexibility<--- Operational performance	1.00	Reference (constrained path)		
SC quality<---Operational performance	.73	.095	7.62	***
SC cost <---Operational performance	.82	.099	8.26	***
SC responsiveness <--- Operational performance	.86	.099	8.69	***
CR1<--- CRM	1.00	Reference		
CR2<--- CRM	.92	.082	11.20	***
IQSP1<--- Status of information sharing	.90	.071	12.72	***
IQSP2 <--- Status of information sharing	1.00	Reference (constrained path)		
IQSP3 <--- Status of information sharing	.85	.075	11.31	***
WIM1 <--- warehouse management	.71	.081	8.67	***
WIM2 <--- warehouse management	.97	.100	9.71	***
WIM3 <--- warehouse management	1.00	Reference (constrained path)		

Note: S.E.=Standard error and ***p-value < 0.001

## Discussion

The exchange of products, information, and funds across channel partners ensures the seamless operation of a supply chain. In practice, before products and cash can flow, information must move downward and upstream in supply chains. Thus, information systems initiate the flow of goods, track them during their movement, confirm their arrival, facilitate payments, and report on the results. As a result, information and communication technologies are vital for collaborating on the ever-increasing information needs of pharmaceutical supply chains [48, 49].

This research conceptualizes an empirical study on the relationship between ICT, supply chain practices, and operational performance and explores the mediating mechanism of ICT adoption at a pharmaceutical supply agency. According to the survey data, ICT posited a significant positive partial mediating role between supply chain practices and operational performance (β = 0.32) relationships. As a result, well-implemented ICT solutions, besides competent workers, can automate day-to-day supply chain tasks and increase the efficiency of supply chain operations and strategic decision-making processes. They can also give a comprehensive inventory picture, eliminate waste and errors, and boost the agency’s operating flexibility. Thus, identifying the ICT implementation hurdles, putting more effort into installing user-friendly ICT tools, and enabling employees to provide desired outcomes are critical areas for agencies to deploy information technologies in a pharmaceutical supply chain auspiciously. These results are comparable to research undertaken in Kenya, Iran, and India in the pharmaceutical sector [6, 50, 51].

Furthermore, we investigated the relationship between PSC practices and operational performance. And it was discovered that there is a significant positive relationship (β = 0.62) between them, demonstrating that better pharmaceutical supply chain practices lead to better supply chain performance. The customer relationship management component had the highest variance (β = 0.78), and working on it improves supply chain performance significantly. A one-unit improvement in CRM enhances operational performance by 78%. Indeed, the agency should implement a strong customer relationship management strategy in its core competencies by regularly analyzing customer satisfaction and understanding consumers’ expectations and requests. Improved PSC practices are becoming increasingly important in measuring and improving agencies’ operational performance by lowering overall operating costs, improving product quality, and developing a culture of flexibility and responsiveness [6, 52]. Despite differences in study constructs, data analysis methods, study settings, and populations, this study substantiates previous research results that emphasized the significant relationship between efficient and effective PSC practices and operational performance improvement in firms [52–55].

The present study indicated that PSC practices (such as CRM, warehousing and inventory management, and the state of information sharing practices) and ICT adoption can predict the operational performance at pharmaceutical supply institutions. Information and communication technology and supply chain practices accounted for 73% of the variation in supply chain performance. The percentage (73%) is remarkable since other factors such as work environment, capital, equipment, and transportation efficiency can impact an organization’s supply chain performance. It suggests that organizations that can manage supply chain practices and ICT adoption effectively may outperform others in terms of operational performance under varied financial, equipment, and transportation conditions.

## Conclusion

The study identified that supply chain practices and ICT implementation affect the supply chain performances of the agency positively and significantly, with supply chain practices having more predictive power than ICT implementation. On the other hand, the ICT implementation practices posited a partial mediating role between supply chain practice and operational performance. The findings suggest that organizations should prioritize and invest in their supply chain practices to enhance their overall supply chain performance. These could involve optimizing warehouse and inventory management, harnessing communication and information sharing, and fostering strong customer relationships. Moreover, the findings indicate that while ICT implementation can have a positive effect on supply chain performance, it is not the only factor that contributes to better performance. As a result, supply chain firms should not rely solely on ICT implementation to improve their supply chain performance but should also focus on optimizing their supply chain practices. Generally, the findings suggest that the firms need to take a holistic approach to supply chain management, considering the supply chain practices and ICT implementation, to achieve better supply chain performance. This has important implications for supply chain managers and decision-makers who should be aware of the importance of the factors and strive for continuous improvement in operational performances. Overall, the study will contribute to a better understanding of the apparent statistical relationships between ICT, supply chain activities, and pharmaceutical supply system performance in Ethiopia. In particular, it provides practical implications for EPSA and pertinent supply chain companies to develop more effective and focused supply chain strategies. For example, the results indicate that customer relationship management and the status of information sharing had a dominant effect on operational performance (Fig. 2). Hence, the agency can invest resources and automate these activities as a top priority. However, the current study is not without limitations, as it only examines the role of digital technology in supply chain practices and performances within a public supplier. Additionally, essential supply chain variables, such as strategic partnership and outsourcing practices, were excluded during the analysis to improve the model’s fit with the data. Thus, prospective researchers can consider these variables and use similar or alternative approaches to investigate the effects of supply chain system automation on the performance of pharmaceutical manufacturers, hospitals, private wholesalers, distributors, and importers.

## Electronic supplementary material

Below is the link to the electronic supplementary material.

Supplementary Material 1. Reliability test results for constructs and sub-constructs

Supplementary Material 2. Rotated component matrix for ICT, supply chain practices and operational performance

## Data Availability

The dataset generated and/or analyzed during the current study is available upon reasonable request from the corresponding author.

## Abbreviations

AVE: Average Variance Extracted
CRM: Customer Relationship Management
EFA: Exploratory Factor Analysis
EPSA: Ethiopian Pharmaceutical Supply Agency
HCMIS: Health Commodity Management Information System
ICT: Information and Communication Technology
PSCM: Pharmaceutical Supply Chain Management
PSCP: Pharmaceutical Supply Chain Practice
SEM: Structural Equation Modeling
VAF: Variation Accounted For
