# Supplementary material for: Exploring the role of ICT in pharmaceutical supply chain practices and operational performance in Ethiopia: a structural equation modeling approach
Source: BMC Health Serv Res. 2023 Jun 14;23:634. doi: 10.1186/s12913-023-09627-w (PMC10266312; doi:10.1186/s12913-023-09627-w)
Supplement: Supplementary file 1 — Supplementary Material 1. Reliability test results for constructs and sub-constructs [file 12913_2023_9627_MOESM1_ESM.docx]

## Additional file 1. Reliability test results for all constructs and sub-constructs

| Constructs | Sub-constructs | No of items | Cronbach alpha value of each sub-construct | Cronbach alpha range |
| --- | --- | --- | --- | --- |
| Operational performance (OP) | Quality | 2 | 0.703 | [0.703. 0.848] |
|  | Cost | 3 | 0.821 |  |
|  | Responsiveness | 3 | 0.790 |  |
|  | Operational flexibility | 3 | 0.848 |  |
|  | Customer’s satisfaction | 3 | 0.763 |  |
| Supply chain practice (SCP) | Customer’s relationships (CR) | 2 | 0.899 | [0.775, 0.899] |
|  | Status of information sharing (IQSP) | 3 | 0.858 |  |
|  | Strategic supplier’s partnerships (SSP) | 2 | 0.843 |  |
|  | Warehouse and inventory management practice (WIMP) | 3 | 0.775 |  |
|  | Outsourcing practice (OSP) | 2 | 0.798 |  |
| The application of ICT | Degree of ICT usage (DOU) | 2 | 0.721 | [0.721,0.894] |
|  | ICT skill of the staffs/ users (SOS) | 2 | 0.894 |  |
| Total no of sub-constructs/questions | | 31 |  |  |
