# Supplementary material for: Exploring the role of ICT in pharmaceutical supply chain practices and operational performance in Ethiopia: a structural equation modeling approach
Source: BMC Health Serv Res. 2023 Jun 14;23:634. doi: 10.1186/s12913-023-09627-w (PMC10266312; doi:10.1186/s12913-023-09627-w)
Supplement: Supplementary file 2 — Supplementary Material 2. Rotated component matrix for ICT, supply chain practices and operational performance [file 12913_2023_9627_MOESM2_ESM.docx]

Additional file 2.Rotated component of matrix for ICT, supply chain practices and operational performance

| Measuring items | Components | | | | |
| --- | --- | --- | --- | --- | --- |
|  | 1 | 2 | 3 | 4 | 5 |
| CR_1_: The agency frequently measures and evaluates customers’ satisfaction to identify future customer expectations, their needs and wants. |  |  |  |  | .786 |
| CR_2_: The agency has a strategy to regularly measure, disseminate and incorporate satisfaction survey findings into its future overall business planning. |  |  |  |  | .822 |
| IQSP_1_: The agency shares information about logistics cost purchasing, inventory levels, and demand forecasts with its key partners. |  | .780 |  |  |  |
| IQSP_2_: The agency informs its trading partners in advance of changing needs and regularly communicates customer’s future strategic need. |  | .829 |  |  |  |
| IQSP_3_: The agency exercises information sharing practice by involving key supplier/s in planning using well established ICT tools |  | .741 |  |  |  |
| WIMP_1_: All available pharmaceutical products are appropriately documented in the essential drug list and data base of the agency |  |  |  | .793 |  |
| WIMP_2_: The agency has minimum central warehouse turn-around time, wastage rate and improved inventory accuracy rate |  |  |  | .803 |  |
| WIMP_3_: The agency has good records of vital pharmaceuticals availability and warehouse order cycle time |  |  |  | .795 |  |
| DOU_1_: Currently in use ICT tools have a good storage capacity/memory in storing big data in one data base | .851 |  |  |  |  |
| DOU_2_: Currently in use ICT system/tools are user friendly and can easily produce reports. | .846 |  |  |  |  |
| SOS_1_: The agency frequently builds strong staff/end user’s awareness on ICT updates | .651 |  |  |  |  |
| SOS_2_: The agency has sufficient groups of ICT experts who can continuously give support and guidance to end users to improve their ICT skills | .652 |  |  |  |  |
| SCQ: Supply chain quality |  |  | .669 |  |  |
| SCC: Supply chain costs |  |  | .730 |  |  |
| SCR: Supply chain responsiveness |  |  | .640 |  |  |
| SCF: Supply chain flexibility |  |  | .608 |  |  |
